# The effect of Sumac on cardiometabolic parameters in adults: a systematic review and meta-analysis of randomized controlled trials

**DOI:** 10.3389/fnut.2024.1305024

**Published:** 2024-01-30

**Authors:** Amirhosein Jafarpour, Saeedeh Jalali, Masoumeh Akhlaghi, Manoochehr Amin Amlashi

**Affiliations:** ^1^Department of Community Nutrition, School of Nutrition and Food Sciences, Shiraz University of Medical Sciences, Shiraz, Iran; ^2^Department of Nutrition, School of Public Health, Iran University of Medical Sciences, Tehran, Iran

**Keywords:** Sumac, lipid profile, anthropometric indices, glycemic indices, blood pressure, cardiometabolic indices

## Abstract

**Background:**

The current evidence on the effect of Sumac consumption on cardiovascular parameters has produced controversial findings.

**Methods:**

We systematically searched several databases, including PubMed-Medline, SCOPUS, and ISI Web of Science, to find eligible studies until January 2023. Meta-analysis to calculated the weighted mean difference (WMD) and 95 %CI, Sub-group meta-analysis and meta-regression analysis were conducted throughout the study.

**Results:**

16 randomized controlled trials comprising a total number of 1,225 participants were included. The results of meta-analysis revealed that Sumac significantly affected low-density lipoprotein (WMD: −8.66 mg/dL; 95% CI: −14.2, −3.12), high-density lipoprotein (WMD: 3.15 mg/dL; 1.99,4.31), triglycerides (WMD: −11.96 mg/dL; −19.44, −4.48), fasting blood glucose (WMD: −4.15 mg/dL; −7.31, −0.98), insulin (WMD: −1.72; −3.18, −0.25), homeostasis model assessment of insulin resistance (HOMA-IR; WMD: −0.61; −1.22, −0.01), and anthropometric indices (*p* < 0.05). Moreover, the results significantly reduced total cholesterol when the intervention duration was ≥12 weeks (WMD: −8.58 mg/dL; −16.8, −0.37).

**Conclusion:**

These findings suggest that Sumac is potentially an effective complementary intervention to improve cardiometabolic parameters. Thus, patients could utilize Sumac as part of their diet to improve their overall cardiometabolic status.

## 1 Introduction

Cardiovascular diseases (CVDs) are linked to several disorders of the heart and blood vessels, including coronary artery disease, atherosclerosis, hypertension, and heart failure (1). CVDs are known as the leading cause of death worldwide. The incidence of CVDs has risen by 77.12% from 31.31 million in 1990 to 55.45 million in 2019; deaths increased by 53.81% from 12.07 million in 1990 to 18.56 million in 2019 (2).

Blood pressure, poor glycemic control, dyslipidemia, and obesity are significant factors related to CVDs (3–6). LDL cholesterol is regarded as “bad” cholesterol because it may induce plaque accumulation in the arteries, leading to atherosclerosis and CVDs. Oxidized LDL particles in the circulation may accumulate in vessel walls and cause plaque development over time. Plaque formation narrows the arterial lumen, producing blood flow resistance and making it harder for the heart and other organs to function. When plaque becomes unstable, it can cause blood clots and arterial blockage and lead to a stroke or heart attack (7).

Elevated blood glucose is a significant risk factor for CVD (8). It may cause oxidative stress and inflammation, endothelial dysfunction, vascular smooth muscle cell proliferation, and platelet activation, and thus contribute to atherosclerosis and CVDs. Over time, hyperglycemia can lead to the production of advanced glycation end-products, which are linked to inflammation and oxidative stress (9).

Today, experimental investigations and, to a lesser degree, clinical trials have highlighted the use of dietary supplements as an adjuvant in treating many diseases, including those affecting the cardiovascular system (10–13). Around the world, the Sumac plant often grows in subtropical and temperate climates, particularly in Africa, North America, and Southeast Asia (14). It is also commonly used as a spice on Iranian table (15). The phytochemical investigations have revealed that Sumac embodies a profuse pool of phenolic compounds comprising delphinidin, chrysanthemin, myrtillin, tannins, and diverse variants of organic acids, specifically malic acid, citric acid, and tartaric acid (16, 17). These components have shown to possess various properties, including antimicrobial, antifungal, antifibrogenic, antimalarial, anti-inflammatory, antioxidant, antimutagenic, anti-thrombin, antitumorigenic, cytotoxic, hypoglycemic, antiviral and leukopenic properties (17). because of its antioxidant properties, accessibility, and minimal side effects compared to hypocholesterolemia drugs, Sumac might be an optimal option for guarding against cardiovascular risk factors (18).

Some meta-analyses have been conducted to show the Sumac effect on glycemic indices and blood lipids. However, no meta-analysis has assessed the Sumac effect on cardiometabolic risk factors together (19, 20). Thus, we conducted this meta-analysis to evaluate the effect of Sumac consumption on cardiometabolic risk factors for the first time.

## 2 Materials and methods

### 2.1 Search strategy

This meta-analysis was conducted based on the guidelines of the PRISMA statement. The criteria of PICOS were clearly defined (Table 1). We searched the PubMed-Medline, SCOPUS, and ISI Web of Science databases to find English-language randomized controlled trials (RCT) with human participants until August 2023 that analyzed the Sumac effect on cardiometabolic risk factors. This search used the following keywords in the title and abstract: rhus* OR “Anacardiaceae” OR “*Rhus coriaria*” OR Sumac* OR Sumach. The search strategy is provided in Supplementary material S1. In addition, a manual search was conducted through the first 20 pages of Google Scholar, and the references of eligible studies were checked to ensure that no relevant reports were missed. Two investigators (S.J. and M.A.) separately assessed each study. Discrepancies were resolved by discussion with A.J. The protocol of this study was registered in the international prospective register of systematic reviews (PROSPERO) database (registration no: CRD42022352515).^1^

**Table 1:** PICOS criteria for inclusion and exclusion of studies.

Parameter	Criteria
Participant	Human adults
Intervention	Sumac consumption
Comparator	Placebo (product without sumac) administration
Outcomes	Effects on cardiometabolic risk factors
Study design	randomized controlled trials

### 2.2 Study selection

The following studies met the criteria for inclusion: clinical trials (in parallel or cross-over designs), studies with an adequately controlled design where Sumac was the only difference between the control and treatment groups, adult participants (over the age of 18), Sumac consumption for at least 2 weeks, and mean and standard deviation (SD), standard error (SE), or 95% confidence interval (CI) values for baseline and post-trial CVD risk factors for each of intervention and control groups presented. CVD risk factors included lipid profile (total cholesterol, low-density lipoprotein cholesterol [LDL-C], high-density lipoprotein cholesterol [HDL-C], fasting triglycerides), glycemic control (fasting glucose, fasting insulin, glycated hemoglobin [HbA1c], homeostasis model assessment of insulin resistance [HOMA-IR]), blood pressure (systolic blood pressure [SBP], diastolic blood pressure [DBP]), and anthropometric indices (body weight, body mass index [BMI], waist circumference). Non-interventional studies, studies without control or placebo groups, observational studies (case–control, cross-sectional, or cohort designs), and trials with insufficient data on baseline or endpoint cardiometabolic risk variables were excluded. Study selection were not restricted by date or location of the study.

### 2.3 Data extraction

The authors conducted an independent review of qualifying studies, examining the first author’s identity, study location, publication year, RCT design (cross-over or parallel), sample size (both intervention and control groups), participant characteristics (including gender, age, and health status), duration of intervention, the quantity of Sumac ingested, and the means and standard deviations (SDs) of the intended outcomes at baseline, post-intervention, and, or changes between baseline and post-intervention.

### 2.4 Quality assessment

The present study presents a detailed account of the quality assessment of the included studies in Supplementary Table 1. Methodological evaluation of the quality of RCTs was conducted based on the Cochrane risk of bias criteria (21). Two independent authors (M.A and S.J) were responsible for rating each study as having a low, high, or unclear risk of bias, based on potential sources of bias such as blinding of outcome assessment, allocation concealment, blinding of participants and personnel, random sequence generation, incomplete outcome data, selective reporting, and other forms of bias. Any discrepancies were resolved through discussion with a third author (A.J).

### 2.5 Statistical analysis

This meta-analysis was performed through the using of Comprehensive Meta-Analysis (CMA) V2 software. The mean alteration of the parameters mentioned above, along with their corresponding standard deviations (SD), were extracted. The effect sizes were articulated in terms of weighted mean differences (WMDs) and 95% confidence intervals. We have computed the standard deviations of the mean differences utilizing the subsequent formula: SD = square root [(SD pre-intervention)^2^ + (SD post-intervention)^2^ − (2 R × SD pre-intervention × SD post-intervention)], supposing a correlation coefficient (R) = 0.5, If the reported values of our variables were in the median and interquartile range (IQR), the estimation of mean and standard SD values was performed using a pre-defined method (22).

Where only SE was reported, SDs were computed using the subsequent formula: SD = SE × sqrt (n), where n is the number of subjects in each group.

The heterogeneity among the studies was assessed utilizing Cochran’s test (*p* < 0.1) and quantitatively through the I^2^ statistic (I^2^ ≥ 50% indicative of notable heterogeneity across the studies). If a significant heterogeneity was demonstrated, a random-effects model was employed; otherwise, a fixed-effects model was utilized.

The sensitivity analysis was performed utilizing the one study remove (leave-one-out) methodology, whereby a single study was omitted, and the analysis was repeated, to evaluate the influence of any study on the overall effect size. A meta-regression analysis was conducted utilizing the unrestricted maximum likelihood method to examine the connection between the overall effect size and the Sumac dose, as well as the duration of intervention.

The analysis of potential publication bias was conducted through the using of funnel plot asymmetry, Begg’s rank correlation, and Egger’s weighted regression tests. We have utilized the Duval & Tweedie ‘trim and fill’ and ‘fail-safe N’ techniques to make adjustments to the analysis, accounting for the influence of publication bias (23).

### 2.6 Grading the evidence

two authors (M.A. and A.J.) independently utilized the GRADE approach. Evidence reliability was evaluated for limitations, inconsistencies, indirectness, inaccuracies, and publication bias, leading to potential downgrading. Discrepancies between the two evaluators were resolved through discussion. Certainty ratings of “High,” “Medium,” “Low,” and “Very low” were assigned to both sets of evidence based on the mentioned criteria (Supplementary Table 3).

## 3 Results

### 3.1 Search results and trial flow

After searching the databases, a total of 969 RCT records were identified, but 199 of them were duplicate publications and removed. Following an assessment of the title and abstract, 752 studies were excluded from further analysis, leaving only 18 studies to undergo full-text evaluation. After a thorough evaluation, five articles were removed for these reasons: inappropriate design (*n* = 3) and did not report adequate information (*n* = 2). Through hand-search, three more studies were found, and thus 16 investigations with 17 arms were included in the meta-analysis (18, 24–38). The flow chart for the process of the study selection is shown in Supplementary Figure 1.

### 3.2 Study characteristics

The characteristics of the studies are provided in Table 2. Data were collected from 16 eligible studies including 17 arms, comprising 1,225 participants, with 613 individuals in the intervention and 612 in the control group. The mean age of the participants was between 23 and 60 years. All selected studies were conducted between 2014 and 2023. ،the Sumac dose ranged between 1 and 3 g/day, and the duration of intervention varied from 6 to 12 weeks. Three studies were carried out on type 2 diabetic patients (26, 36, 38); two trials included patients with non-alcoholic fatty liver disease; two articles had subjects with metabolic syndrome; two studies were conducted on overweight or obese women with depression; in one article subjects were patients with polycystic ovary syndrome (PCOS); one trial carried out on subjects with hypercholesterolemia; one trial was done on patients with primary hyperlipidemia; one research investigations was undertaken on adults who were overweight/obese; one study was conducted on adults diagnosed with dyslipidemia; one study was performed on hypertensive patients and, in one study subjects were hemodialysis patients who received two different quantities of Sumac dose.

**Table 2:** Characteristic of included studies in meta-analysis.

Study	Country	Status	Sample size	Mean age (years)	BMI (Kg/m^2^)	Women (%)	Design	Duration (week)	Dose (g/day)
(36)	Iran	T2DM	41	46.1	46.1	60.97	P,R,PC,DB	12	3
(38)	Iran	T2DM	41	46.1	46.1	NR	P,R,PC,DB	12	3
(27)	Iran	Hypertension	80	59.76	59.76	47.5	P,R,PC,DB	8	1
(18)	Iran	dyslipidemia	30	45.62	45.62	70	C,R,PC,TB	8	1
(33)	Iran	Overweight	49	45.16	45.16	42.85	P,R,PC,DB	6	1
(31)	Iran	Overweight	62	42.19	42.19	100	P,R,PC,DB	12	3
(30)	Iran	Hyperlipidemia	70	45.32	45.32	60	P,R,PC,DB	6	1
(37)	Iran	hypercholesterolemia	172	58.35	58.35	45.34	P,R,PC,DB	12	1
(28)	Iran	NAFLD	80	41.8	41.8	42.5	P,R,PC,DB	12	2
(34)	Iran	NAFLD	80	41.8	41.8	57.5	P,R,PC,DB	12	2
(26)	Iran	T2DM	58	52.3	52.3	NR	P,R,PC,DB	12	3
(25)	Iran	Hemodialysis	71	NR	23.6	50.7	P,R,PC,TB	12	2
(25)	Iran	Hemodialysis	71	NR	24	39.43	P,R,PC,TB	12	3
(29)	Iran	MetS	47	NR	NR	81.25	C,R,PC,TB	6	1
(24)	Iran	PCOS	75	23	25.53	100	P,R,PC,DB	12	3
(32)	Iran	overweight	60	42.89	32.04	100	P,R,PC,DB	12	3
(35)	Iran	MetS	47	58.7	31.6	NR	C,R,PC,TB	6	1

T2DM, Type 2 Diabetes Mellitus; NAFLD, Non-Alcoholic Fatty Liver Disease; MetS, metabolic syndrome; DB, double-blinded; TB, triple-blind, PC, placebo-controlled; R, randomized; P, Parallel; PCOS, poly cystic ovary syndrome; NR, not reported.

### 3.3 Sumac consumption and FBS

Estimated pooled effects using a random-model showed a significant decrease in fasting blood sugar following consumption of Sumac (WMD = −4.15 mg/dL; 95% CI: −7.31, −0.98; *p* = 0.01; I2 = 53.09%). The sub-group analysis revealed that the dose and duration of the intervention were sources of heterogeneity. Furthermore, the consumption of Sumac significantly decreased the levels of fasting blood sugar only with a dosage of ≥3 g/day and a treatment duration of ≥12 weeks (Table 3).

**Table 3:** Subgroup analyses of sumac supplementation on fasting blood glucose in adults.

	NO	WMD ± CI	*p* value	Heterogeneity		
				*p* heterogeneity	I^2^	*p* between sub-groups
Overall effect	10	−4.15 (−7.31, −0.98)	0.01	0.024	53.09%	
Intervention dose (g/day)						0.991
< 3	5	−2.90 (−8.06, 2.25)	0.27	0.001	77.57%	
≥ 3	5	−5.35 (−8.65, −2.05)	0.001	0.85	0%	
Duration (weeks)						
< 12	4	−0.71 (−3.78,2.34)	0.647	0.959	0%	<0.001
≥ 12	6	−7.66 (−10.18, −5.13)	<0.001	0.367	7.66%	

CI, Confidence interval; WMD, weighted mean differences; NO, number of studies.

### 3.4 Sumac consumption and insulin

The estimated pooled effect of 7 individual studies demonstrates a significant decrease in serum insulin (WMD = −1.72; 95% CI: −3.18, −0.25, *p* = 0.021; I2 = 87.88%). After sub-group analysis based on the dose and duration of the intervention, we did not find the source of heterogeneity. These results indicated visible beneficial effects of Sumac consumption at high dosages (dosage ≥3 g/day) and long durations (weeks ≥12) compared to low dosages and short duration of intervention (Table 4).

**Table 4:** Subgroup analyses of sumac supplementation on insulin in adults.

	NO	WMD ± CI	*p* value	Heterogeneity		
				*p* heterogeneity	I^2^	*p* between sub-groups
Overall effect	7	−1.72 (−3.18, −0.25)	0.021	<0.001	87.88%	
Intervention dose (g/day)						0.576
< 3	3	−2.36 (−7.58, 2.85)	0.37	<0.001	94.6%	
≥ 3	4	−0.85 (−1.68, −0.03)	0.042	0.096	52.77%	
Duration (weeks)						
< 12	2	−0.38 (−4.9,4.12)	0.866	0.011	84.71%	0.448
≥ 12	5	−2.25 (−3.92, −0.57)	0.008	<0.001	89.78%	

CI, Confidence interval; WMD, weighted mean differences; NO, number of studies.

### 3.5 Sumac consumption and HOMA-IR

**Figure 1:**
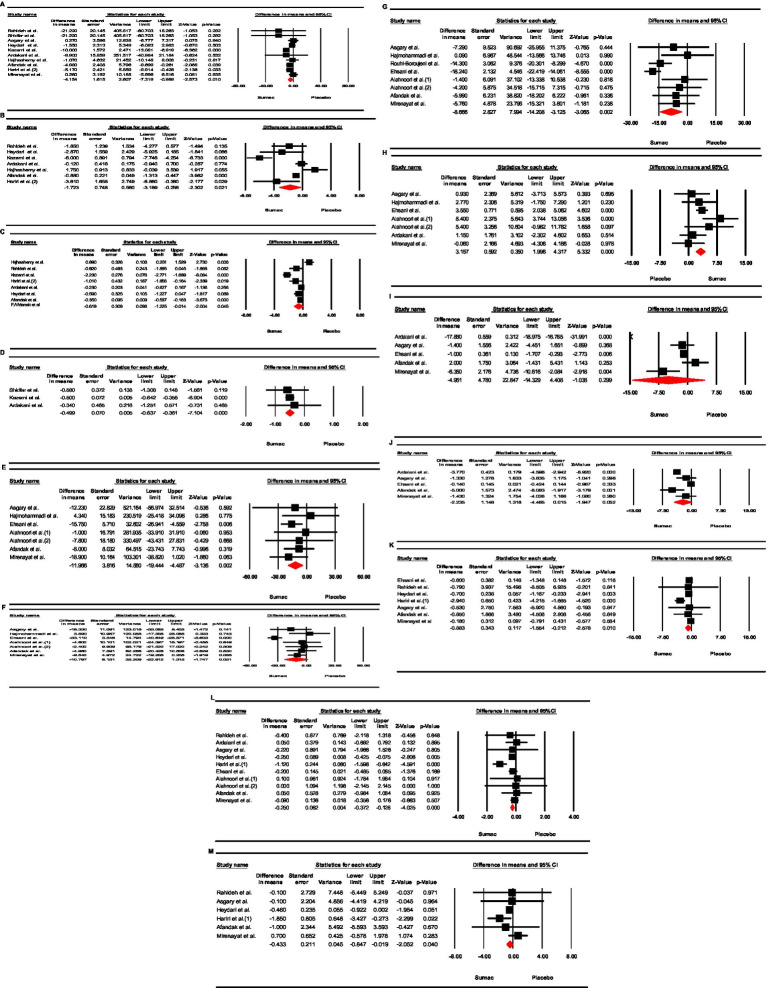
Forest plot detailing weighted mean difference and 95% confidence intervals (CIs) for the effect of garlic supplementation on; **(A)** FBS; **(B)** Insulin; **(C)** HOMA-IR; and **(D)** HbA1c; **(E)** TG; **(F)** TC; **(G)** LDL; **(H)** HDL; **(I)** SBP; **(J)** DBP; **(K)** Weight; **(L)** BMI; **(M)** WC.

Meta-analysis revealed a significant reduction on HOMA-IR levels (WMD = −0.61; 95% CI: −1.22, −0.01, *p* = 0.045; I2 = 90.56%). Upon conducting a sub-group analysis based on the dose and duration of the intervention, the source of heterogeneity was found to be the dosages of interventions. These findings suggest that the consumption of Sumac at high dosage (dosage ≥3gr/day) and for a prolonged duration (weeks ≥12) resulted in a significant decrease in HOMA-IR levels, in opposition to lower dosages and shorter duration of intervention (Figure 1; Table 5).

**Table 5:** Subgroup analyses of sumac supplementation on HOMA-IR in adults.

	NO	WMD ± CI	*p* value	Heterogeneity		
				*p* heterogeneity	I^2^	*p* between sub-groups
Overall effect	7	−0.62 (−1.22, −0.01)	0.045	<0.001	90.56%	
Intervention dose (g/day)						0.792
< 3	3	−0.65 (−2.46, 1.16)	0.48	<0.001	96.3%	
≥ 3	4	−0.4 (−0.64, −0.15)	0.001	0.26	24.33%	
Duration (weeks)						
<12	2	0.15 (−1.30, 1.60)	0.84	0.001	90.33%	0.193
≥12	5	−0.92 (−1.63, −0.21)	0.011	<0.001	91.22%	
					

CI, Confidence interval; WMD, weighted mean differences; NO, number of studies.

### 3.6 Sumac consumption and glycated hemoglobin (HbA1c)

Pooling the effect sizes using a fix-effect model revealed that Sumac had a significant effect in reducing HbA1c (WMD = −0.49, 95% CI: −0.63, −0.36; *p* < 0.001; Table 6).

**Table 6:** Subgroup analyses of sumac supplementation on Hb-A1c in adults.

	NO	WMD ± CI	*p* value	Heterogeneity		
				*p* heterogeneity	I^2^	*p* between sub-groups
Overall effect	3	−0.49 (−0.63, −0.36)	<0.001	0.92	0%	

CI, Confidence interval; WMD, weighted mean differences; NO, number of studies.

### 3.7 Sumac consumption and triglycerides

The estimated pooled effect using a fixed-model showed a significant decrease in triglyceride levels following consumption of Sumac (WMD = −11.96 mg/dL; 95% CI: −19.44, −4.48; *p* = 0.002; I2 = 0). The subgroup analysis indicated that trials administering a Sumac dose of <3 g/day and an intervention period of ≥12 weeks duration resulted in a significant reduction in triglycerides compared to those using higher dosages (dosage ≥3 g/day) or lower durations (<12 weeks; Table 7).

**Table 7:** Subgroup analyses of sumac supplementation on triglycerides in adults.

	NO	WMD ± CI	*p* value	Heterogeneity		
				*p* heterogeneity	I^2^	*p* between sub-groups
Overall effect	7	−11.96 (−19.44, −4.48)	0.002	0.83	0%	
Intervention dose (g/day)						0.524
< 3	5	−13.44 (−22.19, −4.69)	0.003	0.667	0%	
≥ 3	2	−7.96 (−22.36,6.43)	0.278	0.992	0%	
Duration (weeks)						
< 12	2	−0.74 (−25.52, 24.03)	0.95	0.54	0%	0.352
≥ 12	5	−13.09 (−20.93, −5.24)	0.001	0.81	0%	

CI, Confidence interval; WMD, weighted mean differences; NO, number of studies.

### 3.8 Sumac consumption and total cholesterol

Meta-analysis did not detect a statistically significant alteration in plasma total cholesterol concentrations after the administration of Sumac (WMD = −10.29 mg/dL; 95% CI: −22.91, 1.31; *p* = 0.081; I2 = 79.45%). The subgroup analysis revealed that sources of heterogeneity were the duration and dose of the intervention. In opposition to the overall effect, subgroup analysis revealed that trials with intervention durations <12 weeks were effective on total cholesterol (WMD = −8.58 mg/dL; 95% CI: −16.8, −0.37; *p* = 0.04; Table 8).

**Table 8:** Subgroup analyses of sumac supplementation on total cholesterol in adults.

	NO	WMD ± CI	*p* value	Heterogeneity		
				*p* heterogeneity	I^2^	*p* between sub-groups
Overall effect	7	−10.79(−22.91, 1.31)	0.081	<0.001	79.45%	
Intervention dose (g/day)						0.338
< 3	5	−13.36 (−28.27, 1.55)	0.079	<0.001	82.65%	
≥ 3	2	−3.96 (−16.06, 8.13)	0.52	0.84	0%	
Duration (weeks)						
< 12	3	−8.58 (−16.8, −0.37)	0.04	0.415	0%	0.713
≥ 12	4	−12.45 (−31.3, 6.39)	0.195	<0.001	84.83%	

CI, Confidence interval; WMD, weighted mean differences; NO, number of studies.

### 3.9 Sumac consumption and low-density lipoprotein

The estimated pooled effect indicated a significant effect in reducing LDL levels with Sumac consumption (WMD = −8.66 mg/dL; 95% CI: −14.2, −3.12; *p* = 0.002; I2 = 63.97%). The analysis of the subgroups indicated that the potential sources of heterogeneity were the dose and duration of the intervention. Subgroup analysis demonstrated that Sumac consumption can significantly reduce LDL levels at lower doses (< 2 g/day; Table 9).

**Table 9:** Subgroup analyses of sumac supplementation on LDL in adults.

	NO	WMD ± CI	*p* value	Heterogeneity		
				*p* heterogeneity	I^2^	*p* between sub-groups
Overall effect	8	−8.66 (−14.2, −3.12)	0.002	0.007	63.97%	
Intervention dose (g/day)						0.989
< 2	4	−8.56 (−15.31, −1.81)	0.013	0.18	38.72%	
≥ 2	4	−8.47 (−18.2,1.24)	0.087	0.005	76.25%	
Duration (weeks)						
< 12	3	−4.35 (−11.57,2.86)	0.237	0.746	0%	0.208
≥ 12	5	−10.58 (−17.04, −4.12)	0.195	0.013	68.33%	

CI, Confidence interval; WMD, weighted mean differences; LDL, Low density lipoprotein; NO, number of studies.

### 3.10 Sumac consumption and high-density lipoprotein

Meta-analysis using a fixed-effect model showed a significant impact in increasing HDL levels following Sumac consumption (WMD = 3.15 mg/dL; 95% CI: 1.99, 4.31; *p* < 0.001; I2 = 40.12%). When the studies were categorized based on their duration, there was a more significant impact on HDL levels in the subgroup of trials with ≥12 weeks duration (WMD = 3.66 mg/dL; 95% CI: 2.36, 4.96; *p* < 0.001) while trial durations <12 weeks were not effective (WMD = 1.16 mg/dL; 95% CI: −1.41, 3.73; *p* = 0.376). When the studies were classified based on administered Sumac dosage, more significant effect was observed in trials with a dose of ≥2 g/day compared to those with a dosage of <2 g/day (Table 10).

**Table 10:** Subgroup analyses of sumac supplementation on HDL in adults.

	NO	WMD ± CI	*p* value	Heterogeneity		
				*p* heterogeneity	I^2^	*p* between sub-groups
Overall effect	7	3.15 (1.99,4.31)	<0.001	0.124	40.12%	
Intervention dose (g/day)						0.089
< 2	3	1.16 (−1.41,3.73)	0.376	0.66	0%	
≥ 2	4	3.66 (2.36,4.96)	<0.001	0.097	52.51%	
Duration (weeks)						
< 12	3	1.16 (−1.41,3.73)	0.376	0.66	0%	0.089
≥ 12	4	3.66 (2.36,4.96)	<0.001	0.097	52.51%	

CI, Confidence interval; WMD, weighted mean differences; HDL, High density lipoprotein; NO, number of studies.

### 3.11 Sumac consumption and systolic blood pressure

Five studies comprising 195 cases and 194 controls examined the effect of Sumac on systolic blood pressure. The estimated pooled effect using the random-effects model revealed that Sumac had no significant effect on systolic blood pressure (WMD: −4.96 mmHg; 95% CI: −14.32, 4.4; *p* = 0.229; I2 = 99.4%,). Although subgroup analysis was carried out by baseline systolic blood pressure, dose, and duration of the intervention, the source of heterogeneity was not found. Only one trial with a baseline systolic blood pressure of ≥140 (mm/Hg) achieved a significant reduction in systolic blood pressure compared to other trials (Table 11).

**Table 11:** Subgroup analyses of sumac supplementation on systolic blood pressure in adults.

	NO	WMD (95%CI)	*p* value	Heterogeneity		
				*p* heterogeneity	I^2^	*p* between sub-groups
Overall effect	5	−4.96 (−14.32, 4.4)	0.229	<0.001	99.4%	
Baseline SBP (mmHg)						
< 140	4	−1.35 (−3.65, 0.93)	0.246	0.029	66.79%	0
≥ 140	1	−17.88 (−18.97, −16.78)	<0.001	1	0%	
Intervention dose (g/day)						
< 2	3	−8.62 (−20.42, 3.18)	0.152	<0.001	98.3%	0.163
≥ 2	2	0.01 (−2.76, 2.79)	0.994	0.093	64.5%	
Duration (weeks)						
< 12	3	−8.62 (−20.42, 3.18)	0.152	<0.001	98.3%	0.163
≥ 12	2	0.01 (−2.76, 2.79)	0.994	0.093	64.5%	

CI, confidence interval; WMD, weighted mean differences pressure; NO, number of studies.

### 3.12 Sumac consumption and diastolic blood pressure

The estimated pooled effect using the random-effects model indicated that Sumac had no significant impact on diastolic blood pressure (WMD = −2.23 mmHg; 95% CI: −4.48, 0.015; *p* = 0.052; I2 = 94.56%). Results of subgroup analysis revealed that trials with <2 g/day Sumac dose caused a significant reduction in diastolic blood pressure while higher dosages did not. Similarly, Sumac significantly decreased diastolic blood pressure in the subgroup with an intervention duration of <12 weeks with no effect in longer durations (Table 12).

**Table 12:** Subgroup analyses of sumac supplementation on diastolic blood pressure in adults.

	NO	WMD (95%CI)	*p* value	Heterogeneity		
				*p* heterogeneity	I^2^	*p* between sub-groups
Overall effect	5	−2.23 (−4.48, 0.015)	0.052	<0.001	94.56%	
Baseline DBP (mmHg)						
< 80	2	−3.05 (−6.64, 0.53)	0.096	0.07	69.5%	0.591
≥ 80	3	−1.79 (−4.65, 1.07)	0.220	<0.001	96.99%	
Intervention dose (g/day)						
< 2	3	−2.50 (−4.37, −0.64)	0.009	0.061	64.2%	0.942
≥ 2	2	−2.31 (−7.05, 2.41)	0.338	0.002	89.43%	
Duration (weeks)						
< 12	3	−2.50 (−4.37, −0.64)	0.009	0.061	64.2%	0.942
≥ 12	2	−2.31 (−7.05, 2.41)	0.338	0.002	89.43%	

CI, confidence interval; WMD, weighted mean differences; NO, number of studies.

### 3.13 Sumac consumption and weight

The estimated pooled effect using the random-effects approach showed that Sumac had a significant lowering effect on body weight (WMD = −0.88 kg, 95% CI: −1.55, −0.21, *p* = 0.01; I2 = 59.19%). The result of subgroup analysis by duration and dose of the intervention showed that both duration and dosage were potential sources of heterogeneity. When the studies were classified based on the duration of the intervention, there was a significant weight reduction in studies with <12 weeks duration versus those with ≥12 weeks duration (Table 13).

**Table 13:** Subgroup analyses of sumac supplementation on weight in adults.

	NO	WMD ± CI	*p* value	Heterogeneity		
				*p* heterogeneity	I^2^	*p* between sub-groups
Overall effect	7	−0.88 (−1.55, −0.21)	0.01	0.023	59.19%	
Intervention dose (g/day)						0.001
< 3	4	−0.52 (−0.85, −0.19)	0.002	0.614	0%	
≥ 3	3	−2.66 (−3.85, −1.47)	<0.001	0.509	0%	
Duration (weeks)						
< 12	3	−0.50 (−0.87, −0.13)	0.007	0.415	0%	0.026
≥ 12	4	−1.52 (−3.24, 0.2)	0.083	0.022	68.97%	

CI, Confidence interval; WMD, weighted mean differences; NO, number of studies.

### 3.14 Sumac consumption and body mass index

The estimated pooled effect based on fix-effects models indicated a significant BMI reduction (WMD = −0.25 kg/m^2^; 95% CI: −0.37, −0.12; *p* < 0.001; I2 = 41.53%; Table 14).

**Table 14:** Subgroup analyses of sumac supplementation on body mass index in adults.

	NO	WMD ± CI	*p* value	Heterogeneity		
				*p* heterogeneity	I^2^	*p* between sub-groups
Overall effect	10	−0.25 (−0.37, −0.12)	<0.001	0.081	41.53%	
Intervention dose (mg/day)						0.003
< 3	6	−0.19 (−0.32, −0.06)	0.003	0.914	0%	
≥ 3	4	−0.85 (−1.26, −0.43)	<0.001	0.172	39.99%	
Duration (weeks)						
< 12	4	−0.19 (−0.33, −0.05)	0.008	0.706	0%	0.133
≥ 12	6	−0.40 (−0.63, −0.16)	0.001	0.039	57.38%	

CI, Confidence interval; WMD, weighted mean differences; NO, number of studies.

### 3.15 Sumac consumption and waist circumference

Estimated pooled effect using fixed-effects model revealed a significant reduction in waist circumference (WMD = −0.43 cm; 95%CI: −0.84, −0.19; *p* = 0.04) with no significant heterogeneity among the studies (I^2^ = 19.76%). Subgroup analysis by dose and duration of the intervention showed waist circumference decreased substantially in trials with ≥3 g/day intervention dose compared with <3 g/day dosage. Similarly, waist circumference declined significantly in trials with ≥12 weeks duration compared with trials with shorter durations (Table 15).

**Table 15:** Subgroup analyses of sumac supplementation on waist circumference in adults.

	NO	WMD ± CI	*p* value	Heterogeneity		
				*p* heterogeneity	I^2^	*p* between sub-groups
Overall effect	6	−0.43 (−0.84, −0.19)	0.04	0.28	19.76%	
Intervention dose (g/day)						0.085
< 3	3	−0.32 (−0.75, 0.10)	0.142	0.245	28.87%	
≥ 3	3	−1.64 (−3.07, −0.20)	0.025	0.794	0%	
Duration (weeks)						
< 12	3	−0.32 (−0.75, 0.10)	0.142	0.245	28.87%	0.085
≥ 12	3	−1.64 (−3.07, −0.20)	0.025	0.794	0%	

CI, Confidence interval; WMD, weighted mean differences; NO, number of studies.

### 3.16 Sensitivity analysis

The sensitivity analysis has revealed that the calculated overall effect size for systolic blood pressure, BMI, LDL, HDL, and fasting blood glucose levels remained essentially unchanged even after the exclusion of individual studies. However, after the exclusion of investigations conducted by Ehsani et al., the impact of Sumac consumption on diastolic blood pressure and triglyceride levels significantly changed to (WMD = −2.97; 95% CI: −4.53, −1.41) and (WMD = −8.91, 95% CI: −18.96, 1.14), respectively. Moreover, removing studies of Ehsani et al. and Haj Mohammadi et al. changed the overall effect of Sumac on total cholesterol concentration to (WMD = −6.86 mg/dL, 95% CI: −13.29, −0.43) and (WMD = −12.81 mg/dL, 95% CI: −25.48, −0.149), respectively. The exclusion of the research carried out by Heydari et al. and Hariri et al. (1) resulted in an alteration of the overall impact of Sumac on waist circumference to (WMD = −0.32 cm, 95% CI: −1.25, 0.61) and (WMD = −0.32 cm, 95% CI: −0.75, 0.10) respectively. The omission of the investigation conducted by Heydari et al. caused an alteration in the overall influence of Sumac on weight (WMD = −1.04 kg, 95% CI: −2.10, 0.02). Removing the studies by Kazemi et al., Afandak et al., and Hariri et al. changed the overall effect of Sumac on insulin to (WMD: −0.73, 95%CI: −1.73, 0.27), (WMD = −2.02, 95% CI: −4.42, 0.37) and (WMD = −1.51, 95% CI: −3.06, 0.02), respectively. Exclusion of the investigation conducted by Kazemi et al. and Afandak et al. altered the overall impact of Sumac on HOMA-IR to (WMD = −0.3, 95% CI: −0.71, 0.09) and (WMD = −0.67, 95% CI: −1.50, 0.20), respectively.

### 3.17 Meta-regression analysis

Meta-regression was utilized to investigate the potential linear correlation between the dosage and duration of Sumac consumption and cardiometabolic risk factors. The analysis did not reveal any significant correlation between the dose and duration of intervention to alterations in HOMA-IR, insulin, LDL, HDL, systolic blood pressure, diastolic blood pressure, waist circumference, triglycerides, and total cholesterol (Supplementary Table 2). Although BMI and weight did not have any relationship with the duration of the intervention, there was a significant linear correlation between the dose of Sumac consumption and BMI and weight. Furthermore, there was a significant correlation between the dose of the intervention and fasting blood glucose levels, regardless of the duration of the intervention (Supplementary Figure 2).

### 3.18 Publication bias

The results of Begg’s rank correlation test revealed no publication bias in cardiometabolic risk factors. Corrected effect sizes, the result of Egger’s linear regression test, Begg’s rank correlation test, and “fail safe N” tests are added in Supplementary Table 4. Upon visual inspection of the funnel plot, there was no evidence of publication bias in studies that evaluated the effect of Sumac consumption on the cardiometabolic parameters (Supplementary Figure 3).

## 4 Discussion

### 4.1 Main findings

The results of the present study demonstrated the protective effect of Sumac supplementation in improving overall health status. According to the results, Sumac supplementation significantly reduced the levels of triglycerides, LDL, fasting blood glucose, insulin, and HbA1c, decreased HOMA-IR, weight, BMI, and waist circumference, and improved HDL concentration in blood. Regression analysis showed a significant association between the dose of intervention with changes in weight and BMI. The regression analysis results revealed a significant association between the duration of intervention and changes in fasting blood glucose. The Pooled effect size of five studies regarding the effect of the intervention on blood pressure failed to show any significant effect which might be due to the low number of included studies. However, subgroup analysis based on the dosage of intervention revealed that Sumac significantly reduced diastolic blood pressure in studies that utilized lower dosages of intervention (<2 g/day). Based on subgroup analysis, the lowering effect of Sumac on systolic blood pressure is confined to participants with high systolic blood pressure (≥140 mmHg). Re-analyzing studies using sensitivity analysis demonstrated a significant reduction of diastolic blood pressure After omitting a study, However, the results remained insignificant for SBP after stepwise exclusion of each investigation.

### 4.2 Effect of Sumac on lipid profile

In contrast with the present study, a previous meta-analysis failed to demonstrate any significant effect of Sumac supplementation on blood lipid profile which might be due to the low number of included studies (n = 3)in quantitative synthesis (19). Our meta-analysis updated the previous one by pooling the effect sizes of more recent studies. Based on the present study, Sumac supplementation is potentially an effective treatment in improving HDL concentration and reduction of triglycerides and LDL. The mechanism behind this phenomenon is, however, not clearly understood. Several experimental studies have shown the same effect (39–41). There are some possible mechanisms, by which Sumac can improve lipid profile. Gallic acid is a phenolic compound in plants such as Sumac (42). Studies have demonstrated that gallic acid potentially improves lipid metabolism via upregulating metabolic pathways such as β-oxidation of fatty acids and ketogenesis (43). Moreover, gallic acid is shown to reduce LDL, very low-density lipoprotein (VLDL), and triglycerides, and improve HDL concentration in a cardiotoxic-induced animal model (44). Sumac is also rich in phenolic compounds such as quercetin, which modulates the gut microbiome and AMPK/PPAR signaling pathway (45–47). Moreover, kaempferol in Sumac may improve lipid profile by various pathways, including upregulation of hepatic PPARα (48, 49). Our results failed to demonstrate any significant effect of Sumac on total cholesterol concentrations. However, subgroup analysis revealed that Sumac can reduce total cholesterol concentration when the duration of intervention is low (< 12 weeks). Low duration of intervention with Sumac is shown to be not effective on changing concentration of LDL and HDL in the present meta-analysis. However, utilizing Sumac in an extended duration significantly improves concentration of HDL. It might be the factor that Sumac did not change total concentrations of cholesterol in extended duration.

### 4.3 Effect of Sumac on obesity indices

The effect of Sumac supplementation on obesity indices was investigated in the present meta-analysis. There is no meta-analysis to be compared with our results. We found that Sumac supplementation significantly reduced weight, BMI, and waist circumference. Weight reduction is a complex mechanism involving several pathways, including appetite regulation and energy homeostasis (50). Sumac is shown to have a very robust inhibitory effect on pancreatic lipase enzyme activity and thus can reduce fat absorption and calorie intake (51). Moreover, Sumac is shown to be a critical source of bioactive compounds including polyphenols (52) and studies have shown that food-derived phenolic compounds have the capacity of promoting energy expenditure through the activation of brown adipose tissue (53). Quercetin is another possible ingredient in Sumac that can expedite the thermogenesis in brown adipose tissue and increase energy expenditure (46, 54).

Hunger and satiety sensations involve various physiological and biochemical pathways that regulate food intake and energy hemostasis (55). Natural phenolic compounds such as quercetin, kaempferol, gallotannins, and gallic acid have been shown to have appetite suppression properties including reduction of ghrelin, resistin, and glucagon-like peptide-1 (GLP-1) concentrations, and induction of serotonin and leptin pathways (56). Interestingly, Sumac is a natural source of phenolic compounds mentioned above (52).

### 4.4 Effect of Sumac on glycemic control

We expanded the results by conducting a meta-analysis on glycemic indices. A previous meta-analysis conducted to evaluate the effect of Sumac supplementation on glycemic indices failed to show a significant effect of Sumac on glycemic parameters (20). In opposition to previous study Updating the results with more recent trials revealed that Sumac supplementation significantly reduced fasting blood glucose insulin, HbA1c concentrations, and the level of HOMA-IR. Galic acid which is widely found in Sumac is shown to have an inhibitory effect on carbohydrate digestive enzymes including α-amylase and α-glucosidase and thus could lower postprandial glycemic response and eventuality reduce the overall risk in diabetes patients (52, 57, 58). Furthermore, it is found that galic acid upregulates mRNA expression of GLUT-4 and IRS-1 in adipose tissue (59). Moreover, the quercetin content of Sumac could possibly be attributed to improved glucose metabolism in skeletal muscle cells and hepatocytes via stimulating AMPK and thus increased GLUT4 translocation (52, 60). Galic acid is also shown to have a protective effect on pancreatic islet cells via modulation of inflammatory and oxidative pathways also increases the secretion of insulin from the pancreas (61).

### 4.5 Effect of Sumac on blood pressure

We also investigated the effect of Sumac supplementation on blood pressure. We found that the overall effect of Sumac supplementation did not have a significant effect on systolic and diastolic blood pressure. Conducting a subgroup analysis based on baseline levels of blood pressure, dose, and duration of intervention revealed that the lowering effect of the intervention on systolic blood pressure is confined to those participants with high systolic blood pressure (≥140 mmHg) and that on diastolic blood pressure was observed in lower Sumac doses (<2 g/day). Higher dosage of Sumac supplementation results in an insignificant increase in systolic blood pressure. Sumac contains minerals that shown to have protective effect on blood pressure, namely, potassium, calcium, magnesium (62–64), however it also contains sodium that might led to increased blood pressure (65).

### 4.6 Strengths and limitations

The present investigation contained strengths and limitations that should be considered in the overall interpretation of the results. The literature review showed that our study was the first investigation to evaluate the effect of Sumac supplementation on all cardiometabolic factors. Furthermore, we expanded the results by conducting several sub-analyses including sub-group analysis and meta-regression to find the source of heterogeneity. Besides, we comprehensively searched the literature to reduce bias in the review process. However, the low number of included participants in some factors, high heterogeneity and failure to find the source of the heterogeneity, lack of enough arms to conduct subgroup analysis, and risk of bias in some factors should be considered as limitations of the study in the final interpretation. Additionally, Sumac might hold cultural, historical, or traditional significance in Iran. Consequently, while studies have been conducted exclusively in Iran, conducting research across multiple countries could provide insights into the universality of these findings.

### 4.7 Clinical and public health implications

Several investigations demonstrated the beneficial effect of complementary therapies via plant-based intervention to have a positive effect on cardiometabolic indices. We found that Sumac is one of these interventions that could be used in the medical setting. Patients could benefit from the results of the present study as clinicians might include Sumac supplementation as a complementary therapy besides conventional intervention to improve overall cardiometabolic status in patients.

## 5 Conclusion

We found that Sumac especially at higher duration could potentially improves glycemic parameters including fasting blood glucose, fasting insulin concentrations, HbA1c and HOMA-IR levels especially at higher dosages and higher duration of intervention. Moreover, we found that Sumac could potentially aid in controlling lipid profile by lowering the concentrations of triglycerides and LDL and improving HDL concentrations. Anthropometric indices including body wight, BMI, and waist circumference potentially improves by utilizing Sumac daily. Thus, this study gives an insight to a potential planed based intervention to improve cardiometabolic disturbances. Clinicians could advice participants with cardiometabolic disturbances to take Sumac supplementation for aiding in overall health status. More trials with high duration and high dosage of Sumac are advised to be assess Sumac supplementation on other cardiometabolic parameters including inflammation and oxidative stress.

## Data availability statement

The original contributions presented in the study are included in the article/Supplementary material, further inquiries can be directed to the corresponding author.

## Author contributions

AJ: Conceptualization, Data curation, Formal analysis, Investigation, Methodology, Software, Visualization, Writing – original draft. SJ: Data curation, Investigation, Writing – original draft. MA: Supervision, Validation, Writing – review & editing. MAA: Project administration, Supervision, Validation, Writing – review & editing.

## Figures and Tables

**Table:** 

AMPK	Adenosine monophosphate-activated protein kinase
BMI	body mass index
CMA	Comprehensive Meta-Analysis
CVDs	Cardiovascular diseases
DBP	diastolic blood pressure
FBS	fasting blood sugar
GLP-1	glucagon-like peptide-1
GLUT-4	Glucose transporter type 4
HbA1c	Hemoglobin A1C
HDL-C	high-density lipoprotein
HOMA-IR	homeostasis model assessment of insulin resistance
IRS-1	Insulin receptor substrate-1
LDL-C	low-density lipoprotein
PCOS	polycystic ovary syndrome
PPAR	Peroxisome proliferator-activated receptors
SBP	systolic blood pressure
TC	total cholesterol
TG	triglycerides
VLDL	very low-density lipoprotein
WC	waist circumference
WMD	weighted mean difference

## Footnotes

^1^ http://www.crd.york.ac.uk/PROSPERO
