# Supplementary material for: The effect of Sumac on cardiometabolic parameters in adults: a systematic review and meta-analysis of randomized controlled trials
Source: Front Nutr. 2024 Jan 30;11:1305024. doi: 10.3389/fnut.2024.1305024 (PMC10861764; doi:10.3389/fnut.2024.1305024)
Supplement: Supplementary file 1 [file Data_Sheet_1.docx]

Studies screened (n=827)

Full text article for eligibility (n=18)

Screening

Eligibility

Included

Studies excluded after duplicates removal (n=203)

Records identified after databases search (n=1030)

Identification

809 articles were excluded after evaluating the title and abstract.

**Records excluded (n=5)**

Inappropriate design (n=3)

Unavailable in full text (n=2)

Hand searched(n=3)

Studies included in quantitative synthesis (meta-analysis): (n=16)

**Supplementary figure 1.** Flowchart of study selection for inclusion trials in the systematic review.

1. **FBS C) HOMA-IR**

1. **Insulin D) TG**

**Supplementary figure 2.** Random-effects meta-regression plots of the association between dose of sumac (mg/day) and weighted mean difference of A) FBS; and B) Insulin; C) HOMA-IR; D) TG.

**E) TC G) HDL-C**

**F) LDL-C H) SBP**

**Supplementary figure 2.** Random-effects meta-regression plots of the association between dose of sumac (mg/day) and weighted mean difference of E) TC; and F) LDL-C; G) HDL-C; H) SBP

1. **DBP K) BMI**

1. **Weight L) WC**

**Supplementary figure 2.** Random-effects meta-regression plots of the association between dose of sumac (mg/day) and weighted mean difference of I) DBP; and J) Weight; K) BMI; L) WC

1. **FBS C) HOMA-IR**

1. **Insulin D) TG**

**Supplementary figure 2.** Random-effects meta-regression plots of the association between duration of intervention and weighted mean difference of A) FBS; and B) Insulin; C) HOMA-IR; D) TG.

**E) TC G) HDL-C**

**F) LDL-C H) SBP**

**Supplementary figure 2.** Random-effects meta-regression plots of the association between duration of intervention and weighted mean difference of E) TC; and F) LDL-C; G) HDL-C; H) SBP

1. **DBP K) BMI**

1. **Weight L) WC**

**Supplementary figure 2.** Random-effects meta-regression plots of the association between duration of intervention and weighted mean difference of I) DBP; and J) Weight; K) BMI; L) W

1. **FBS C) HOMA-IR**

1. **Insulin D) Hb-A1C**

**Supplementary figure 3.** Funnel plot for the effect of sumac consumption on A) FBS; B) Insulin; C) HOMA-IR; and D) Hb-A1C.

1. **TG G) LDL-C**
2. **TC H) HDL-C**

**Supplementary figure 3.** Funnel plot for the effect of sumac consumption on E) TG; F) TC; G) LDL-C; and H) HDL-C.

1. **SBP K) Weight**

1. **DBP L) BMI**

**M) WC**

**Supplementary figure 3.** Funnel plot for the effect of sumac consumption on I) SBP; J) DBP; K) Weight; L) BMI and M) WC.

**Supplementary table 1.** Risk of bias for randomized controlled trials, assessed according to the Revised Cochrane risk-of-bias tool for randomized trials (RoB 2).

| Study | Random sequence generation | Allocation concealment | Selective reporting | Other sources of bias | Blinding (participants and personnel) | Blinding (outcome assessment) | Incomplete outcome data | General risk of bias |
| --- | --- | --- | --- | --- | --- | --- | --- | --- |
| Rahideh et al. | L | L | U | L | L | U | L | Some concerns |
| Asgary et al. | L | L | U | L | L | U | L | Some concerns |
| Shidfar et al | U | U | U | L | L | U | L | High risk of bias |
| Ardalani et al. | L | U | U | L | L | U | L | High risk of bias |
| Heydari et al. | U | U | U | L | L | U | L | High risk of bias |
| Hariri et al. | U | U | U | H | U | U | L | High risk of bias |
| Hajmohammadi et al. | U | U | U | H | L | H | L | High risk of bias |
| Rouhi-Boroujeni et al. | U | U | U | H | L | H | L | High risk of bias |
| Ehsani et al. | U | U | U | H | L | H | L | High risk of bias |
| Kazemi et al | U | U | U | H | L | H | L | High risk of bias |
| Fatahi Ardakani et al | U | U | U | H | L | H | L | High risk of bias |
| Hajhashemy et al | L | U | U | L | L | U | L | High risk of bias |
| Alahnoori et al | L | U | U | L | L | U | L | High risk of bias |
| Afandak et al | L | L | U | L | L | U | L | Some concerns |
| Hariri et al. | L | U | U | H | L | H | L | High risk of bias |
| Mirenayat et al. | U | L | U | L | L | U | L | Some concerns |

| **Model** | **Fixed effect** | | | **Random effect** | | |
| --- | --- | --- | --- | --- | --- | --- |
| - | slope | 95%CI | P-value | slope | 95%CI | P-value |
| **Fasting blood sugar** |  |  |  |  |  |  |
| Dose of consumption | -0.002 | -0.006,0 | 0.09 | -0.002 | -0.006,0 | 0.09 |
| Duration of intervention | -1.21 | -1.87, -0.54 | **0.0004** | -1.21 | -1.87, -0.54 | **0.0004** |
| **Insulin** |  |  |  |  |  |  |
| Dose of consumption | -0.0002 | -0.002,0,001 | 0.81 | -0.0002 | -0.002,0,001 | 0.81 |
| Duration of intervention | -0.34 | -0.92,0.22 | 0.23 | -0.34 | -0.92,0.22 | 0.23 |
| **HOMA-IR** |  |  |  |  |  |  |
| Dose of consumption | -0.0003 | -0.001,0.0005 | 0.5 | -0.0003 | -0.001,0.0005 | 0.5 |
| Duration of intervention | -0.17 | -0.4,0.05 | 0.12 | -0.17 | -0.4,0.05 | 0.12 |
| **Systolic blood pressure** |  |  |  |  |  |  |
| Dose of consumption | 0.005 | -0.002,0.014 | 0.19 | 0.005 | -0.002,0.014 | 0.19 |
| Duration of intervention | 1.62 | -1.69,4.95 | 0.33 | 1.62 | -1.69,4.95 | 0.33 |
| **Diastolic blood pressure** |  |  |  |  |  |  |
| Dose of consumption | -0.0008 | -0.003,0.002 | 0.63 | -0.0008 | -0.003,0.002 | 0.63 |
| Duration of intervention | -0.01 | -0.91,0.89 | 0.98 | -0.01 | -0.91,0.89 | 0.98 |

**Supplementary table 2 Meta-regression between changes in cardio metabolic risk factors and administered doses and intervention duration of sumac**

**Supplementary table 3. GRADE profile of folic acid supplementation for SBP and DBP**

| Quality assessment | | | | | | Summary of findings | | Quality  of evidence |
| --- | --- | --- | --- | --- | --- | --- | --- | --- |
| Outcomes | Risk of bias | Inconsistency | Indirectness | Imprecision | Publication Bias | Number  of intervention/control | WMD (95%CI) |
| FBS | Serious | serious | not serious | not serious | not serious | 315/304 | -4.15 (-7.31, -0.98) | ⨁⨁◯◯ |
| insulin | serious | serious | not serious | not serious | not serious | 216/208 | -1.72 (-3.18, -0.25) | ⨁⨁◯◯ |
| HOMA-IR | serious | serious | not serious | not serious | not serious | 216/208 | -0.62 (-1.22, -0.01) | ⨁⨁◯◯ |
| HbA1c | serious | not serious | not serious | not serious | not serious | 92/87 | -0.49 (-0.63, -0.36 | ⨁⨁⨁◯ |
| triglycerides | serious | not serious | not serious | not serious | not serious | 258/263 | -11.96 (-19.44, -4.48) | ⨁⨁⨁◯ |
| TC | serious | serious | not serious | serious | not serious | 258/263 | -10.79(-22.91, 1.31) | ⨁◯◯◯ |
| LDL | serious | serious | not serious | not serious | not serious | 344/349 | -8.66 (-14.2, -3.12) | ⨁⨁◯◯ |
| HDL | serious | not serious | not serious | not serious | not serious | 258/263 | 3.15 (1.99,4.31) | ⨁⨁⨁◯ |
| SBP | serious | serious | not serious | serious | not serious | 195/194 | -4.96 (-14.32, 4.4) | ⨁◯◯◯ |
| DBP | serious | serious | not serious | serious | not serious | 195/194 | -2.23 (-4.48, 0.015) | ⨁◯◯◯ |
| weight | serious | serious | not serious | not serious | not serious | 234/227 | -0.88 (-1.55, -0.21) | ⨁⨁◯◯ |
| BMI | serious | not serious | not serious | not serious | not serious | 341/342 | -0.25 (-0.37, -0.12) | ⨁⨁⨁◯ |
| WC | serious | not serious | not serious | not serious | not serious | 194/180 | -0.43 (-0.84, -0.19) | ⨁⨁⨁◯ |

| **Fall safe N test** | **Egger’s linear regression test** | | | | | **Begg’s rank**  **correlation test** | | | **Corrected**  **effect size** | |  |
| --- | --- | --- | --- | --- | --- | --- | --- | --- | --- | --- | --- |
| n | p-value | df | t | 95%CI | Intercept | p-value | z-value | Kendall’s  Tau | 95%CI | WMD |  |
| 42 | 0.64 | 8 | 0.48 | -1.6,2.4 | 0.42 | 0.32 | 0.98 | -0.25 | -7.06,  -0.83 | -3.94 | FBS |
| 49 | 0.43 | 5 | 0.85 | -5.97, 2.99 | -1.49 | 0.36 | 0.9 | -0.28 | -1.8, 1.3 | -0.27 | Insulin |
| 62 | 0.59 | 5 | 0.56 | -7.5,4.8 | -1.36 | 0.54 | 0.6 | -0.19 | -1.2,  -0.1 | -0.61 | HOMA-IR |
| 20 | 0.84 | 1 | 0.25 | -4.32, 4.49 | 0.08 | 1 | 0 | 0 | -0.63,  -0.36 | -0.49 | Hb-A1C |
| 16 | 0.93 | 8 | 0.08 | -1.58, 1.48 | -0.05 | 0.72 | 0.35 | -0.08 | -0.38, -0.14 | -0.26 | BMI |
| 0 | 0.96 | 4 | 0.04 | -2.05, 1.97 | -0.03 | 1 | 0 | 0 | -0.84, -0.01 | -0.43 | WC |
| 22 | 0.53 | 5 | 0.66 | -3.2,1.9 | -0.66 | 1 | 0 | 0 | -1.98, -0.43 | -1.2 | Weight |
| 4 | 0.18 | 5 | 1.51 | -0.55, 2.1 | 0.8 | 0.76 | 0.3 | 0.09 | -21.02, -7.13 | -14.08 | TG |
| 37 | 0.05 | 5 | 2.56 | -0.01, 7.6 | 3.7 | 1 | 0 | 0 | -24.9, -4.4 | -14.65 | TC |
| 68 | **0.001** | 6 | 5.73 | 1.78,4.4 | 3.11 | 0.38 | 0.86 | 0.25 | -19.12, -8.83 | -13.98 | LDL |
| 31 | 0.86 | 5 | 0.17 | -3.02, 2.63 | -0.19 | 0.54 | 0.6 | 0.19 | 1.99,  4.31 | 3.15 | HDL |
| 360 | 0.9 | 3 | 0.13 | -37.74, 34.74 | -1.5 | 0.8 | 0.24 | -0.1 | -15.1, 1.73 | -6.68 | SBP |
| 56 | 0.26 | 3 | 1.35 | -11.03, 4.43 | -3.3 | 0.46 | 0.73 | -0.3 | -4.48, 0.01 | -2.23 | DBP |

**Supplementary table 3 Assessment of publication bias in the impact of sumac on cardio metabolic risk factors**
